# Whiskey’s Work in Illinois

**Published:** 1912-06

**Authors:** 


### Whiskey's Work in Illinois.

Some few months ago Herbert Spring, a young man of Rockford, left his home in that city and went to Freeport, about twenty-eight miles distant. According to his testimony about the first thing that he did was to go into a saloon and get a drink. He was a minor, but he got his drink just the same. And then a little later on, according to his testimony, he had quite a number of drinks. The result, of course, was that he became insanely intoxicated. According to Rufus M. Potts, attorney for the Rusch Brewing Co., he was getting in proper condition of mind to properly celebrate with full appreciation the Birth and Resurrection of Christ, Fourth of July, Decoration Day, etc.

On the afternoon of that day Mrs. Edna Rumelhagen started down town to do some shopping. She was pushing before her a baby carriage, in which her six-months'-old baby sat cooing. Little did this young mother and proud mistress of her home dream of the fate that awaited her. Without any warning whatever she was set upon by the above-named drunken boy, who, in his imaginary delirium of intoxication, began to fire at her the contents of his revolver, and finally one of the bullets pierced her heart and she dropped dead.

The young lad was arrested and lodged in jail, where, after he had slept off his drunken debauch, declared that he knew nothing whatever of his movements. But that did not change the situation. Over in the Rumelhagen home the young husband was prostrated with grief and the motherless infant cried for the young mother that lay cold in death. The result, we can say, directly and positively of intoxicating liquor.

Last week the young man was tried, pleaded guilty and sentenced to a life imprisonment at hard work in the Joliet penitentiary. The sentence pronounced by Judge Farnand is rather remarkable and we print it in full. It contains some awful warning and some splendid advice. The warning, as in most such cases, however, came too late to prevent the prison doors closing forever upon the life of a young man not yet twenty-one. It brought small comfort or consolation to his heart-broken father and mother, who sat weeping and crushed under the awful tragedy. But it may suffice to cause other young men who are becoming reckless to pause and think. The sentence is as follows:

"During the past five years seven men have been before me charged with murder; four have pleaded guilty, three have been tried by jury. In each case the defendant was under the influence of intoxicating liquor at the time the offense charged was committed, as you were when you committed the crime to which you have entered the plea of guilty.

"Intoxication can not be considered as a defense in this case. The young man who at your age has become a frequenter of saloons and an habitual drinker of intoxicating liquors is almost certain of moral death before he reaches mature manhood. Oh, could I speak to the young men of this nation I would beg of them to shun the saloon; let not its shadows fall upon you; they will blight and finally destroy your young manhood, bring unutterable sorrow to those who love you, and hasten you prematurely to the grave or to a home within the prison walls.

"Two years ago at Dixon a young man of your age stood before me to receive his sentence on a plea of guilty to the crime of murder. What I said to him then I want to say to you now. There are two great lights which should always control the court in a case of this nature—first, justice; second, humanity. I have given much thought in my efforts to determine what justice demands; what humanity asks, in this case. We can not bring back the dead; a life has been taken and the lifeless body of one has been laid in the grave, and if it comes again it must be only when God commands that the earth and sea give up their dead. We are to deal alone with the living.

"During the progress of this trial there has been a picture constantly before us which has caused the court to think deeply of the tragedy of life and the mystery and sorrow of death. Upon one side the mother and father, widower and motherless child of the one who sleeps; upon the other the mother, father and other members of your family; all sorrowing, some for the dead, others for the living; and it is difficult to determine which of the sorrowing ones suffer the greatest grief. All this has come because of your cruel act. But through and beyond the dark clouds of sorrow and the tears of grief the court must look alone to the evidence and there search for the light which leads to the right.

"You are a young man, just having passed your twentieth year. In law you have not yet passed from the scenes of childhood to the more dignified and responsible one of manhood. Yet you have mental powers sufficient to discern and follow the right and to know and fully understand the consequences of such a crime as you have committed. She whom you shot to death was younger than you, yet for a brief time she had borne that almost sacred name 'mother.' Her heart, which beat in love for her first-born, was pierced by a bullet fired from a weapon in your hand, the breast that gave nurse to her infant child was chilled and she slept.

" 'My God, my baby, I am dying,' were the agonizing and pathetic words which fell from the lips of your victim just before she passed into the valley where the shadows of life faded away and the twilight of eternity broke upon her. Those words will at times ring in your ears until the morn of that day when you, too, must pass into that valley, and if the light which then breaks upon you from the further shore is radiant with hope it will be only because an offended but all-wise and merciful God has forgiven you and your awful sin.

"Under the sentence I am about to impose you shall have ample time while in prison to think of the past and meditate on the future. Day by day, week by week, month by month and year by year, until the sands have run out of the dial of your life, you shall wear the garb of a prisoner; the narrow walls of the criminal's cell shall be your abiding place. When the iron gates close behind you the beauties of life and the grandeur of nature shall be hidden from you, and home and mother will be only memories. But let them be sweet memories. Remember the teachings and virtues of that mother whose heart is bleeding with agony today. Her love will follow and bless you in your darkest hour. I sometimes feel that a mother's love has in it a spark of divinity. I know not the time nor place when or where the love of a pure mother will not enfold her child. Under the influence of that love prepare yet to live according to her teachings and her prayers. There is a God in heaven whose infinite power and tender mercy can and will penetrate the gloom of a prison cell. His power enables Him to search and know the human heart. You have transgressed His laws. He alone can give you solace in the coming years of grief.

"It is the sentence of this court that you be imprisoned in the penitentiary at Joliet for and during the term of your natural life, the first day of that imprisonment to be in solitary confinement, the balance of the time at hard labor, and that you pay the costs of prosecution." If this had been your boy, how would you vote?—*Exchange.*

---

IF WE WANT pure food, cold storage conditions regulated, mosquitoes, flies, rats and other carriers of fevers and plague exterminated; if we want the spread of tuberculosis, meningitis, and children's diseases stopped; if we want child labor laws, limitations of hours of women's work, proper health conditions in our public schools, then we should favor a national department of health.—Senator Robert M. La Follette.

---

If a hernia suddenly becomes irreducible, advise prompt operation; if the patient vomits, even once, insist upon it!—*American Journal of Surgery.*

